# Squirrelpox Virus: Assessing Prevalence, Transmission and Environmental Degradation

**DOI:** 10.1371/journal.pone.0089521

**Published:** 2014-02-21

**Authors:** Lisa M. Collins, Neil D. Warnock, David G. Tosh, Colin McInnes, David Everest, W. Ian Montgomery, Mike Scantlebury, Nikki Marks, Jaimie T. A. Dick, Neil Reid

**Affiliations:** 1 Quercus, School of Biological Sciences, Queen’s University Belfast, Belfast, Northern Ireland; 2 School of Biological Sciences, Queen’s University Belfast, Belfast, Northern Ireland; 3 Institute for Global Food Security (IGFS), Queen’s University Belfast, Belfast, Northern Ireland; 4 School of Life Sciences, University of Lincoln, Lincolnshire, England; 5 Moredun Research Institute, Penicuik, Midlothian, Scotland; 6 Animal Health and Veterinary Laboratories Agency (AHVLA), Addlestone, Surrey, England; USGS National Wildlife Health Center, United States of America

## Abstract

Red squirrels (*Sciurus vulgaris*) declined in Great Britain and Ireland during the last century, due to habitat loss and the introduction of grey squirrels (*Sciurus carolinensis*), which competitively exclude the red squirrel and act as a reservoir for squirrelpox virus (SQPV). The disease is generally fatal to red squirrels and their ecological replacement by grey squirrels is up to 25 times faster where the virus is present. We aimed to determine: (1) the seropositivity and prevalence of SQPV DNA in the invasive and native species at a regional scale; (2) possible SQPV transmission routes; and, (3) virus degradation rates under differing environmental conditions. Grey (*n* = 208) and red (*n* = 40) squirrel blood and tissues were sampled. Enzyme-linked immunosorbent assay (ELISA) and quantitative real-time polymerase chain reaction (qPCR) techniques established seropositivity and viral DNA presence, respectively. Overall 8% of squirrels sampled (both species combined) had evidence of SQPV DNA in their tissues and 22% were in possession of antibodies. SQPV prevalence in sampled red squirrels was 2.5%. Viral loads were typically low in grey squirrels by comparison to red squirrels. There was a trend for a greater number of positive samples in spring and summer than in winter. Possible transmission routes were identified through the presence of viral DNA in faeces (red squirrels only), urine and ectoparasites (both species). Virus degradation analyses suggested that, after 30 days of exposure to six combinations of environments, there were more intact virus particles in scabs kept in warm (25°C) and dry conditions than in cooler (5 and 15°C) or wet conditions. We conclude that SQPV is present at low prevalence in invasive grey squirrel populations with a lower prevalence in native red squirrels. Virus transmission could occur through urine especially during warm dry summer conditions but, more notably, via ectoparasites, which are shared by both species.

## Introduction

Red squirrels (*Sciurus vulgaris*) have been in decline in Great Britain for the last century due to a combination of habitat loss and the introduction of the North American eastern grey squirrel (*Sciurus carolinensis*) [1]. Grey squirrels are believed to act as direct competitors to red squirrels for habitat and resources and appear to be able to out-compete them [2]–[4]. Additionally, both species are susceptible to the squirrelpox virus (SQPV), a member of the poxviridae with some genomic similarity to Orf virus in sheep [5].

In grey squirrels, SQPV is a sub-clinical infection that rarely manifests in disease [6]–[7]. However, in red squirrels it causes ulceration with crusted lesions and scabs around the eyes, lips, feet and genitalia, and an exudative dermatitis which may be mis-diagnosed due to other similar looking diseases [8], yet is almost always fatal [9]–[10]. Epidemiological studies using the enzyme-linked immunosorbent assay (ELISA) method for detecting the presence of SQPV-specific antibodies have demonstrated a high presence of SQPV antibodies in healthy grey squirrels, yet reported an absence of SQPV antibodies in otherwise healthy red squirrels [6]. These findings indicate that grey squirrels are a natural reservoir for SQPV and are capable of resisting the disease, whilst the red squirrel does not appear to have this capability [9], [11]–[12]. Red squirrel populations in close proximity to SQPV seropositive grey squirrels have been reported to decline up to 25 times faster than those in close residential proximity to seronegative grey squirrels [4]. However, a recent investigation has revealed the presence of SQPV antibodies in live red squirrels [13]. This finding provides hope for the sustainability of a viable red squirrel population, but also the development of a SQPV vaccine [11]. Despite this, SQPV is nevertheless considered one of the major contributing factors in the decline of red squirrels throughout the UK and seriously threatens current conservation efforts [4].

Reports of pox-like infection of red squirrels in the British Isles first appeared in the early 1900 s [14]. However, the first confirmed case of SQPV in a red squirrel was in 1981 [15]. Since then, there have been multiple outbreaks across the UK. The disease was not known in Ireland until 2011, when two separate incidents were recorded in Northern Ireland (County Down, March 2011 and County Antrim, June 2011) [16]. More recently, there was a confirmed case in the Republic of Ireland (County Wicklow, November 2011; Sean Callanan, *unpublished data*). These recent cases of SQPV in red squirrels reflect increasing seropositivity in grey squirrels and emergent cases in red squirrels throughout Ireland [16]. It is expected, therefore, that as the grey squirrel continues to expand its range, contact rates between the two species will rise, increasing the likelihood of SQPV spreading to remaining red squirrel populations [17].

To date, ELISA has been the predominant method used in epidemiological studies for the detection of SQPV. However, this represents an incomplete picture of SQPV occurrence as it tests only for the presence of antibodies (an indicator of past infection and potentially current infection status) but cannot provide an indication of current infection rates. Information on both the presence of virus particles (as assessed by detection of viral DNA) and their concentrations in red and grey squirrel populations is necessary to understand the spread of the disease. Quantitative real-time polymerase chain reaction (qPCR) can be used to determine the presence of viral DNA in squirrel tissue [18].

SQPV transmission routes from grey to red squirrels and from red to red squirrels have not been explored. It has been hypothesised that direct contact between infected animals, the shedding of pox virons into the environment and the involvement of ectoparasites as vectors are the primary means of transmission [12], [16], [18]. Developmental work in these areas is required to understand SQPV epidemiology to predict levels of risk to remaining red squirrel populations and to inform future conservation efforts.

The principal objectives of this study were to: (i) use ELISA and qPCR to assess both SQPV seropositivity and viral DNA presence in grey and red squirrels; (ii) analyse saliva, urine, faeces and ectoparasites from SQPV positive squirrels for the presence of SQPV DNA, to investigate their potential as a means of disease transmission; and, (iii) analyse virus degradation rates under six environmental conditions: a factorial combination of ‘wet’ or ‘dry’ conditions at three temperatures (5, 15 and 25°C).

## Methods

### Ethics Statement

This work was approved by the Queen’s University Belfast, School of Biological Sciences, Research Ethics Committee on 20/02/2012. Grey squirrels were trapped using baited Albion Manufacturing ALBI 079 traps, which were checked twice daily. As some forests were known to have both red and grey squirrels present, a trapping license was obtained (license number TSE/8/12) from the Northern Ireland Environment Agency (NIEA). Trapped grey squirrels were euthanized by cranial concussion, following [19]. Red squirrels are a protected species and were not culled as part of this study. Instead, forest rangers and members of local squirrel conservation groups were asked for any carcasses found dead (through squirrel-vehicle collisions, predation or natural death), or suspected infected cases culled as part of an NIEA disease control strategy. The forests used in this study were all public areas.

### Sample Collection

Grey squirrels were trapped between March and June 2012. At each site, 10 cage-traps of 175×150×600 mm (Albion Manufacturing; ALBI 079 squirrel trap), and the area surrounding the trap, were baited with peanuts, maize and sunflower seeds following previously published recommendations [20]. Traps remained at each site between 2 and 8 days (mean = 4.4 days). Any trapped red squirrels were released.

Culled grey squirrels (April 2010–June 2012) were also supplied by Northern Ireland Forest Service Rangers, members of local Red Squirrel Conservation Groups and members of the public. All carcasses were frozen and stored at −20°C and defrosted prior to tissue harvesting and processing. Each squirrel was sexed.

### Sampling Candidate Routes of Transmission

Saliva was collected from 12 qPCR positive grey squirrels and the sole qPCR positive red squirrel and extracted from their defrosted carcasses. Samples were collected using a double swab technique to maximise DNA recovery [21]. Two swabs, the first dipped in sterile water, the second dry, were rolled over each side of the buccal cavity and tongue in a circular fashion with moderate pressure.

A section of the lower intestinal tract was removed from the same 12 qPCR positive grey squirrels and the positive red squirrel as for the saliva samples. Faecal samples from individual squirrels were separated from the intestinal wall using a scalpel to minimise contamination of non-shed intestinal wall.

The bladders of 133 grey squirrels (of both positive and negative SQPV status) were removed and examined for the presence of urine. A total of 38 squirrels (all of which were SQPV negative) appeared to have urine present, which was extracted by clamping the bladder using arterial forceps and removing it from the carcass using a scalpel. Urine was extracted by piercing the bladder using a 23 gauge 31.75 mm needle.

Ectoparasites from six qPCR positive grey squirrels and one positive red squirrel were tested for SQPV. Each parasite was identified as a flea, tick or mite.

### ELISA Analysis

Squirrel blood was extracted from each carcass using a 23 gauge 31.75 mm needle and sent to Moredun Research Institute (Midlothian, Scotland) for enzyme-linked immunosorbent assay (ELISA) analysis to test for the presence of SQPV antibodies. ELISA methodologies were those previously published [5]. Results were categorised as positive if corrected OD values were ≥0.2 or negative if <0.2.

### DNA Extraction

DNA from a 25 mg segment of the lower lip from each squirrel and from whole ectoparasite samples was extracted using the DNeasy Blood and Tissue kit (QIAGEN) and eluted in 100 µl of elution buffer following the manufacturer’s instructions. DNA was extracted from saliva and urine samples using the QIAamp DNA Micro Kit (QIAGEN) following manufacturer’s instructions. DNA from faecal material samples was extracted using TRIzol Reagent (Invitrogen) following manufacturer’s instructions. DNA equivalent to 1 mg of lip tissue, 1 µl of blood, 1/10 of total ectoparasite DNA or 1/4 of saliva and urine DNA was added to each PCR reaction. Published forward and reverse SQPV primers described in [18] were used to detect SQPV DNA. qPCR analysis was performed on the Bio-Rad CFX96 C1000 using Fast start SYBR Green Master (Roche) according to manufacturer’s instructions, under the following cycling conditions: (95°C×10 min, 40×(95°C×10 s, 65°C×10 s, 72°C×20 s) 72°C×10 min [18]. To ensure primer specificity and that amplicons were of the correct length, products were analysed by melt curve analysis and checked on a 0.4% agarose gel containing 0.0075% ethidium bromide from a 10 mg/ml stock.

qPCR protocols, including sensitivity and specificity of the reaction, were as developed and optimised [18]. Samples were classed ‘positive’ if 2 of 3 replicates had a CT lower than 35 cycles, that was significantly different from the negative and no template controls, together with a melt of 87–88°C. On those occasions where negative controls were positive, the sample with an additional negative control was repeated. Quantification of the viral load was determined by the standard curve method using a serial dilution of a SQPV standard [18]. Virus load in squirrel lip and faeces was calculated using a standard diluted in negative squirrel DNA and negative faeces DNA respectively to account for observed inhibition in amplification. Virus load in blood, ectoparasites, saliva and urine was calculated using the standard diluted in water, as no PCR inhibition was observed. The standard was produced from the reaction of SQPV forward and reverse qPCR primers on a positive squirrel sample, the resulting amplicon was gel extracted, cloned using ‘Qiagen PCR cloning kit’ following manufacturer’s instructions and sequenced using the ‘Dundee Sequencing Service.’ Data presented on viral DNA copy number is the inferred number of virus particles present in a milligram of tissue (v/mg), present in one ml of blood (v/ml), a whole ectoparasite specimen (v/e), swabbed saliva (v/s) and one ml of urine (v/ml) deduced from genomic equivalents of the starting material.

### Virus Degradation Analysis

**Figure 1:**
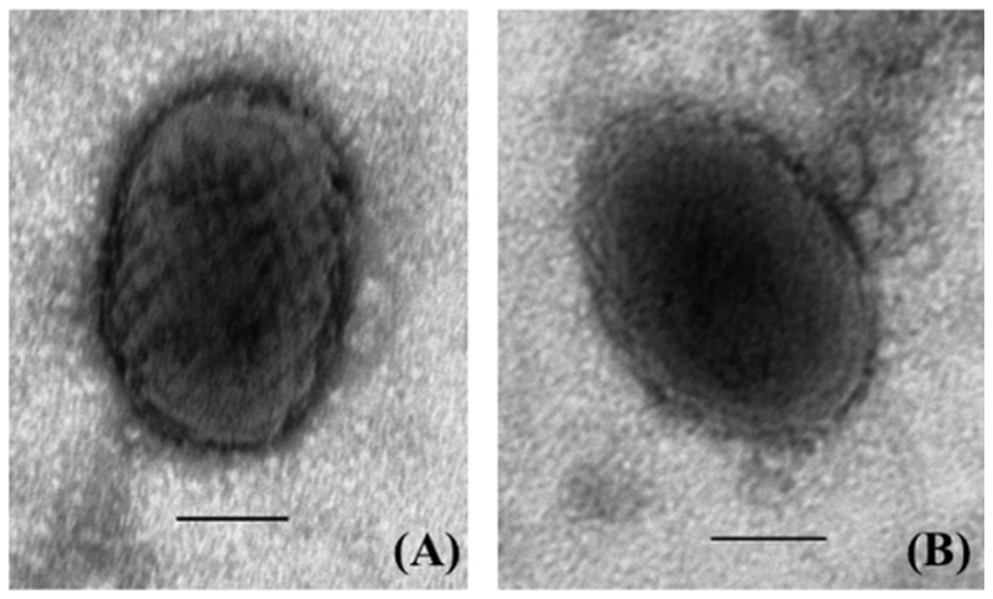
Negative contrast stain Transmission Electron Microscopy (TEM) images of squirrelpox virons. (**A**) Intact virons with surface morphology and (**B**) degraded virons lacking surface morphology.

The viability of the virus in the environment was investigated using a factorial combination of (i) wet or dry conditions and (ii) three temperatures (5, 15 and 25°C) for a period of 30 days, giving a total of six treatment groups. A total of 15 replicates were repeated in each experimental treatment. A section of scab from an infected red squirrel (donated by Prof. Sean Callanan, University College Dublin) was used as a source for virus. The scab was dried over silica gel for a period 36 hours in a dessicator [22], sectioned into 1 mg pieces using a scalpel with 1 mg placed in separate Eppendorfs. Dry condition Eppendorfs were sealed in an airtight polythene zipper bag with silica gel crystals placed at the bottom. This bag was in turn sealed in a larger airtight polythene zipper bag. For the wet condition Eppendorfs, a volume of distilled water roughly equal in volume to the amount of dried scab in each Eppendorf was added to samples. These wet samples were sealed in an airtight polythene zipper bag with a damp cloth at the bottom. This bag was in turn sealed in a larger airtight polythene zipper bag. After a period of 30 days, samples were analysed by Negative Contrast Stain Transmission Electron Microscopy (TEM) for the presence of identifiable virus particles [23]. The numbers of degraded (lacking surface morphology) and intact (with surface morphology) particles (Figure 1) were enumerated.

### Statistical Analyses

Descriptive cross-tabulation was used to assess the sensitivity and specificity of the qPCR and ELISA techniques for capturing the disease status of red and grey squirrels. Percentage values for SQPV antibody or DNA prevalence within the sample of squirrel tested are given as exact figures, however, estimates of percentage prevalence within localities, species or seasons (i.e. were our sample has been used to make inferences about the wider population) are accompanied with 95% confidence intervals (±95%CIs) generated by 1000 iteration resample bootstrapping using the Resampling Stats for Excel (RSXL) add-in Version 4 (downloaded from www.resample.com/excel).

The occurrence of positive results for: a) qPCR; b) ELISA; and c) overall disease status (both test results combined) were examined using Generalized Linear Mixed Models (GLMMs) assuming a binomial error structure and a logit link function. Locality (Forest) was fitted as a hierarchical nested random factor to account for multiple squirrels collected from each forest within each locality. Disease status (infected or uninfected) was fitted as the dependent variable. Variance was examined with respect to Locality, Species, Sex, the occurrence of Ectoparasites and Season.

The number of degraded and intact virus particles detected by TEM remaining after 30 days of exposure to simulated environmental conditions was analysed using Generalized Linear Models (GLMs) assuming a Poisson error structure (for count data) and a logarithmic link function. Temperature (5, 15 and 25°C) and Condition (wet or dry) were fitted as categorical factors, including a two-way interaction.

Maps were generated using ArcMap 10.1 (ESRI, California, USA). The dataset for this study is freely available to other researchers from the corresponding author.

## Results

**Figure 2:**
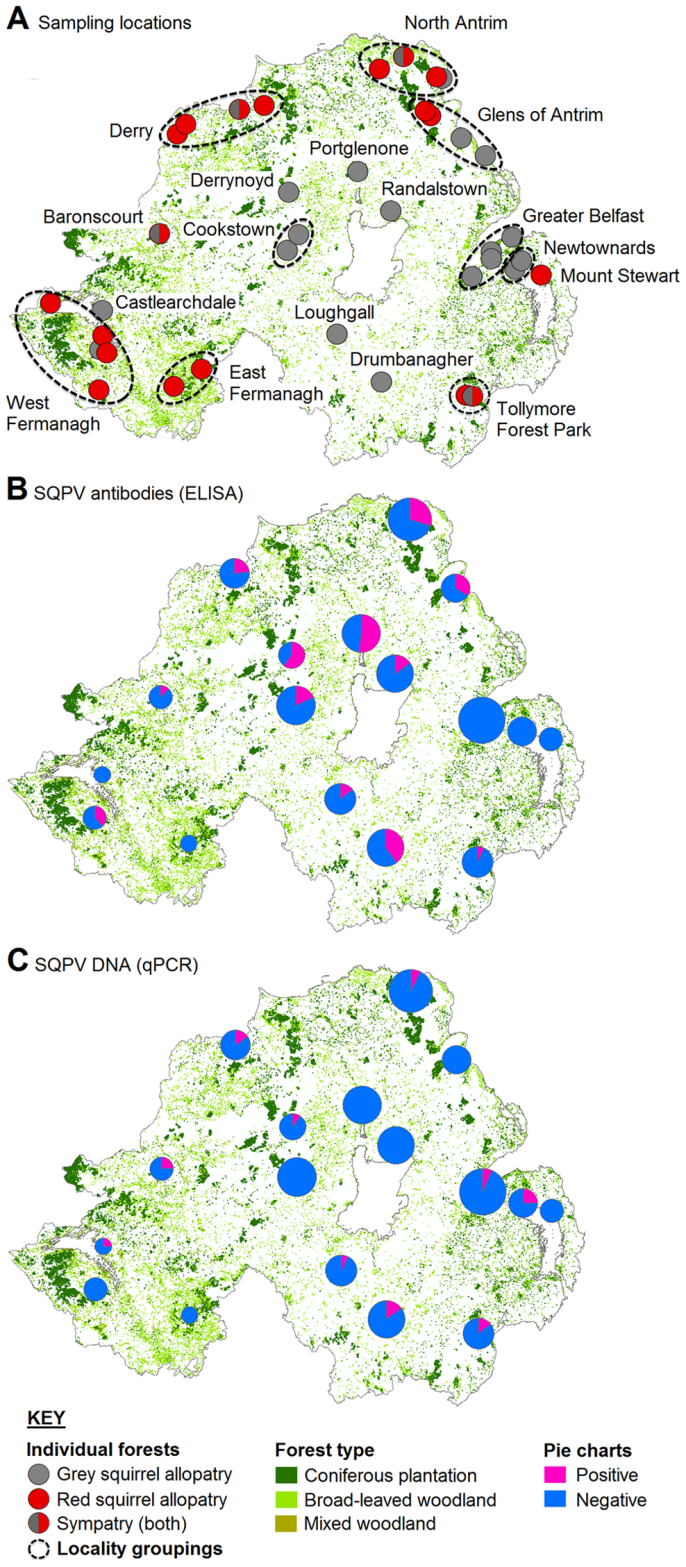
Maps of sampling effort and results. (**A**) Location of individual forests (*n* = 37) grouped into localities (*n* = 17) from which squirrel carcasses were collected. (**B**) The prevalence of squirrelpox antibodies as determined by ELISA. (**C**) The prevalence of squirrelpox DNA as determined by qPCR. The size of each pie chart is scaled for sample size i.e. total number of squirrels tested in each locality.

Grey squirrel (*n* = 208) and red squirrel (*n* = 40) carcasses were collected (total *n* = 248) from April 2010 to June 2012 (Table 1) from 37 individual forests within 17 localities throughout Northern Ireland (Figure 2A). Eleven animals that were supplied by conservation groups conducting grey squirrel culling could not have the season in which they were culled accurately attributed and were therefore not included in analyses, which reduced sample sizes; spring (*n* = 143), summer (*n* = 57), autumn (*n* = 4) and winter (*n* = 33).

**Table 1:** Capture rates of grey and red squirrels in allopatric populations (either grey squirrels only or red squirrels only) or sympatric populations (mixed).

		Grey squirrels		Red squirrels	
Population status	No. of forests	Total number	Mean per forest ± s.e.	Total number	Mean per forest ± s.e.
Grey only	18	164	9.1±1.5	0	0.0±0.0
Red only	15	0	0.0±0.0	29	1.9±0.5
Mixed	4*	44	11.0±3.1	11^†^	3.7±0.9^†^
***Total***	***37***	***208***	***9.5±1.3***	***40***	***2.2±0.4***

*Of the four forests known to have a mixed population, red squirrels were sampled at only three sites which ^†^accounts for the mismatch between the mean number of red squirrels and the total numbers caught.

### ELISA and qPCR

Fifty four (22%) of 248 squirrels sampled (both species combined) tested positive for SQPV antibodies using the ELISA technique compared to 19 (8%) testing positive for viral DNA using the qPCR technique on lip tissue and 4 (2%) of 243 squirrels sampled using qPCR on blood. The results of ELISA and lip tissue qPCR matched in 74% of cases (Table 2), ELISA and blood qPCR in 77% of cases (Table 3), ELISA and the combined results from both lip and blood in 72% of cases (Table 4) and lip and blood qPCRs in 92% of cases (Table 5). Only four squirrels (2%) tested positive for both antibodies and viral DNA in either the lip tissue or blood.

**Table 2:** Descriptive cross-tabulation of blood ELISA and lip tissue qPCR results.

	Lip qPCR		
ELISA	*−ve*	*+ve*	Total
***−ve***	179 (72)	15 (6)	194 (78)
***+ve***	50 (20)	4 (2)	54 (22)
**Total**	229 (92)	19 (8)	248 (100)

Values represent the numbers of squirrels (of both species combined) with percentages in parentheses.

**Table 3:** Descriptive cross-tabulation of blood ELISA and qPCR results.

	Blood qPCR		
ELISA	*−ve*	*+ve*	Total
***−ve***	188 (77)	3 (1)	191 (79)
***+ve***	51 (21)	1 (<1)	52 (21)
**Total**	239 (98)	4 (2)	243 (100)

**Table 4:** Descriptive cross-tabulation of blood ELISA and combined lip and blood qPCR results.

	Combined qPCR		
ELISA	*−ve*	*+ve*	Total
***−ve***	177 (71)	17 (7)	194 (78)
***+ve***	50 (20)	4 (2)	54 (22)
**Total**	227 (92)	21 (8)	248 (100)

**Table 5:** Descriptive cross-tabulation of lip and blood qPCR results.

	Lip qPCR		
Blood pPCR	*−ve*	*+ve*	Total
***−ve***	222 (91)	17 (7)	239 (98)
***+ve***	2 (1)	2 (1)	4 (2)
**Total**	224 (92)	19 (8)	243 (100)

Fifty three (25%) of 208 grey squirrels sampled tested positive for antibodies. Prevalence varied significantly between localities (Table 6) from zero (undetected) to 60±30% i.e. mean ±95%CI (Figure 2B). By comparison, twenty grey squirrels sampled (10%) tested positive for virus DNA using either lip tissue or blood. The relatively low variation in the prevalence of viral DNA meant that it did not vary significantly between localities (Table 7) where prevalence ranged from zero (undetected) to 25±25% (Figure 2C) with the wide 95% confidence interval being the result of low statistical power. Only 3 grey squirrels sampled (1%) tested positive for both antibodies and viral DNA.

**Table 6:** Generalized Linear Mixed Model (GLMM) results for blood ELISA results (*F*_df = 22,212_ = 1.762, *p* = 0.022, AUC = 0.875).

Parameter	*F*	n.df	d.df	*p*
Locality	1.926	16	212	0.020
Species	6.827	1	212	0.010
Sex	2.593	2	212	0.109
Ectoparasite presence	0.108	1	212	0.743
Season	1.638	4	212	0.182

**Table 7:** GLMM results for qPCR results i.e blood and lip combined (*F*_df = 22,212_ = 0.667, *p* = 0.869, AUC = 0.858).

Parameter	*F*	n.df	d.df	*p*
Locality	0.684	16	212	0.809
Species	0.542	1	212	0.463
Sex	0.039	2	212	0.844
Ectoparasite presence	0.004	1	212	0.953
Season	0.675	4	212	0.568

One (3%) of 40 red squirrels sampled tested positive for both antibodies and viral DNA. This individual was collected at Tollymore Forest Park, Co. Down (Figure 2A) which was one of the four forests supporting sympatric populations i.e. grey and red squirrels together. Prevalence within Tollymore Forest Park red squirrels, of which 5 were tested, was 20±40%.

Viral loads (representative of the amount of virus genome present) in lip tissue were low for infected grey squirrels (median = 63 v/mg). One individual had a moderately high load of 4901 v/mg. In comparison, the viral load of the only positive red squirrel was almost 50,000 times higher than the median viral load for grey squirrels at 3,113,957 v/mg. Only three grey squirrels and one red squirrel tested positive for SQPV DNA in their blood (grey squirrel median = 8,370 v/ml and red squirrel median = 21,032,512 v/ml).

**Figure 3:**
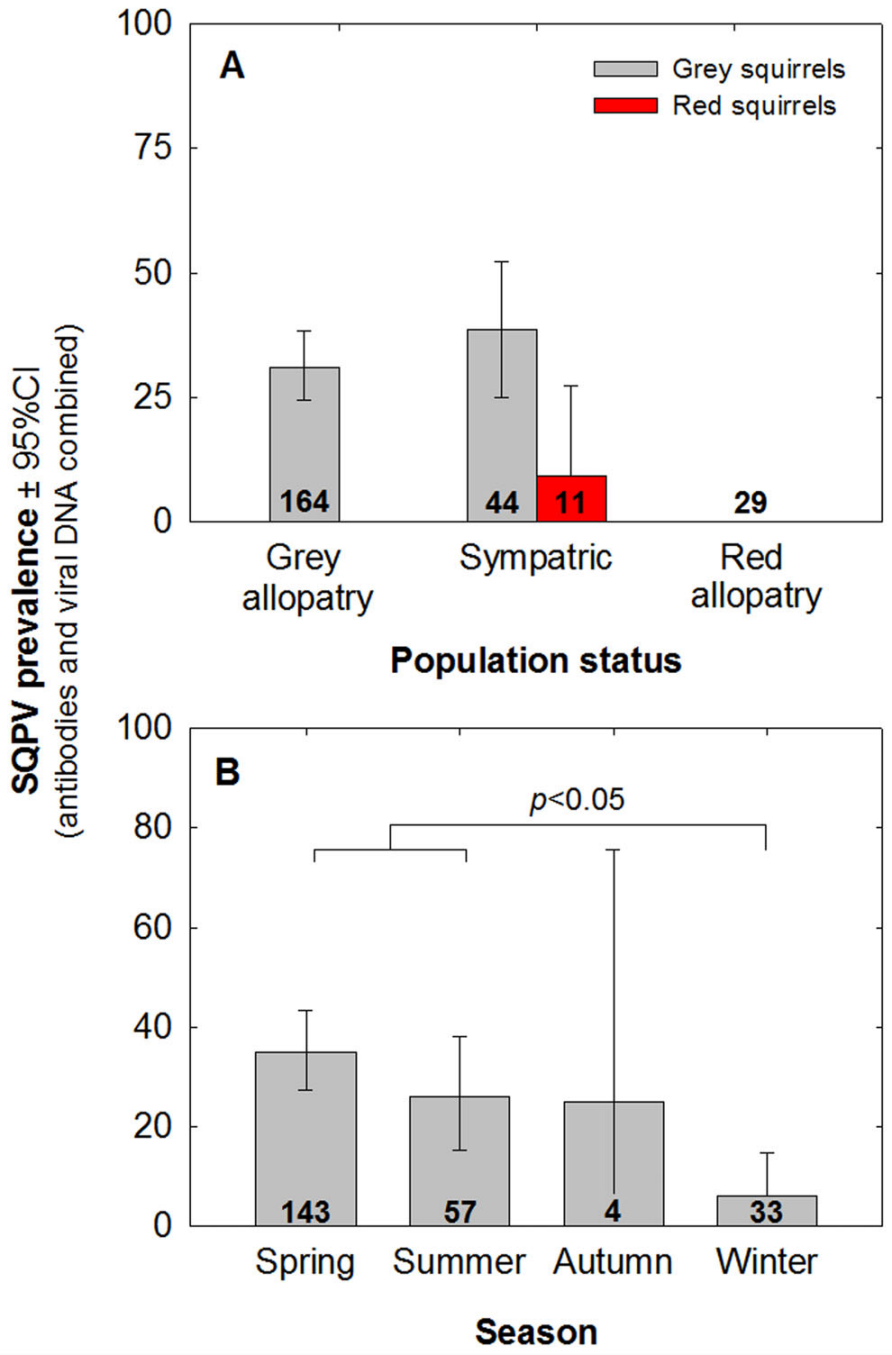
Squirrelpox prevalence. The effect of (**A**) species and (**B**) season for overall disease status i.e. percentage presence of antibodies from ELISA and/or viral DNA from qPCR ±95%CIs. Sample sizes (*n*) are shown in the bars.

There were no statistical correlates of the presence of viral DNA in tissues as determined by qPCR (Table 7). The presence of antibodies and overall disease status (i.e. the presence of antibodies plus viral DNA) varied significantly between species (Table 8) with prevalence being greater in grey squirrels (Figure 3A). Overall disease status also varied significantly between seasons (Table 8) with prevalence being significantly higher in both spring and summer than winter (Figure 3B). SQPV was widespread in squirrels throughout Northern Ireland (Figure 2B & C).

**Table 8:** GLMM results for overall disease status i.e. blood ELISA and blood and lip pPCR results combined (*F*_df = 22,212_ = 1.775, *p* = 0.021, AUC = 0.838).

Parameter	*F*	n.df	d.df	*p*
Locality	1.824	16	212	0.030
Species	8.121	1	212	0.005
Sex	1.338	2	212	0.249
Ectoparasite presence	0.054	1	212	0.816
Season	2.724	4	212	0.045

### Routes of Transmission

Saliva samples taken from only qPCR positive grey squirrels were all negative for viral DNA (under limit of detection). The single positive red squirrel had a high level of SQPV DNA in its saliva (29,549,584 v/s). Lip tissue and blood of 38 grey squirrels from which urine was extracted were all negative for SQPV. However, the urine from two (5%) of the 38 squirrels sampled which tested positive for viral DNA had a viral load of 1040 v/ml and 880 v/ml respectively.

All faecal samples from qPCR positive grey squirrels appeared normal and were negative for SQPV when also analysed by qPCR. Faeces from the infected red squirrel was black in colour (which may have indicated the presence of blood) and liquid in appearance suggesting diarrhoea. This sample contained a moderate level of SQPV (18,434 v/s).

Ectoparasites occurred on 144 (69%) of 206 grey squirrels sampled and 9 (23%) of 40 red squirrels sampled (note two of the 208 grey squirrel carcasses were not examined for ectoparasites due to their condition). Fleas were the most abundant parasite, found on 69% of grey squirrels and 18% of red squirrels sampled. Ticks (*Ixodes ricinus*) were present on 2% of grey squirrels and 13% of red squirrels sampled, whilst mites occurred on 3% of grey squirrels sampled but were not detected on any sampled red squirrels. All parasites collected from the infected red squirrel tested positive for SQPV DNA using qPCR, while 27% of the fleas tested from SQPV DNA positive grey squirrels had virus present. Viral loads were relatively low for ectoparasites on grey squirrels (median = 25 virus particles per ectoparasite (v/e), *n* = 11) in comparison to ectoparasites on the one infected red squirrel (median viral load: fleas = 2,874 v/e, *n* = 3; ticks = 2,162 v/e, *n* = 3; other = 25,683 v/e, *n* = 3). Any ectoparasites found on squirrels that were negative for SQPV DNA were also negative for SQPV DNA.

### Virus Degradation

**Figure 4:**
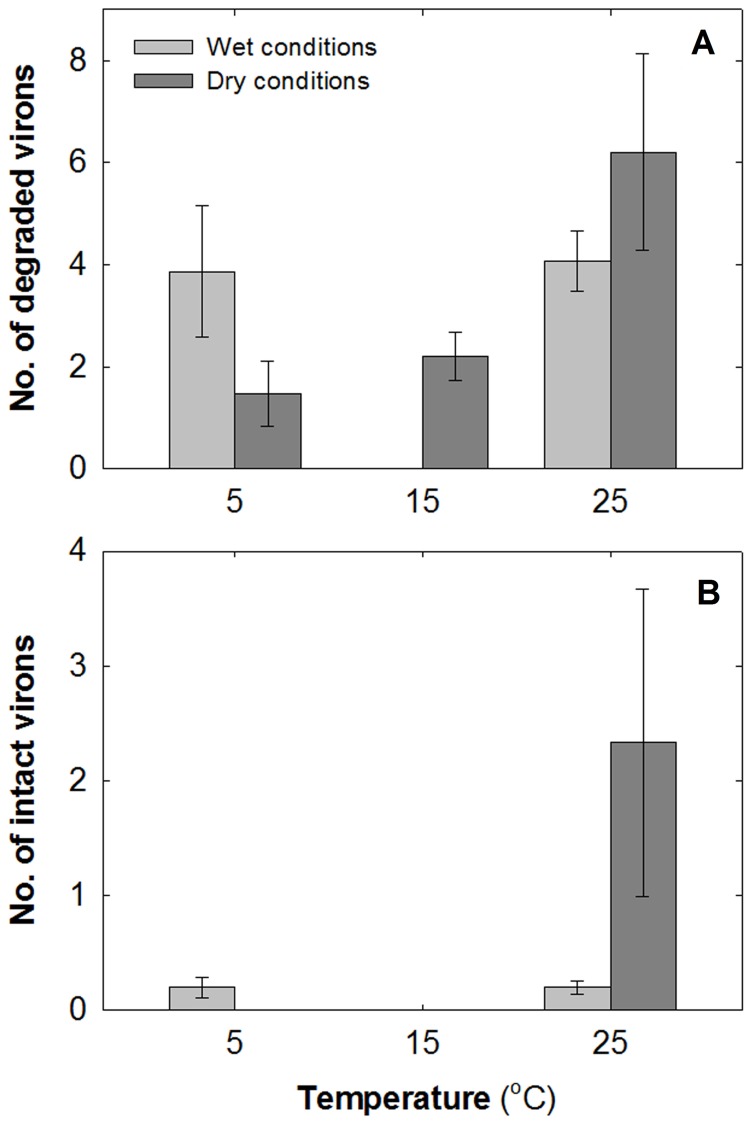
Mean number of particles ±1 standard error. (**A**) Degraded and (**B**) intact virus particles in wet and dry conditions at three temperatures (*n* = 15 replicates in each category) remaining after one month of exposure. Note that no degraded particles were detected at 15°C in wet conditions and no intact particles at 15°C in either condition.

After 30 days, the number of degraded virus particles differed significantly between temperatures under both wet and dry conditions (Wald χ^2^ = 21.54, df = 1, *P*<0.0001). More degraded virus particles were observed in scabs kept in warm (25°C), dry conditions than cooler (5 or 15°C) or wet conditions (Figure 4A). The number of intact virus particles at the end of the 30 day exposure period varied significantly between temperatures (Wald χ^2^ = 17.92, df = 1, *P*<0.0001) and wet/dry conditions (Wald χ^2^ = 15.93, df = 1, *P*<0.0001). In dry conditions, intact virus particles remained only at the highest temperature (25°C). In wet conditions, there were few intact particles at 5 and 25°C and none at 15°C (Figure 4B).

## Discussion

The location of allopatric and sympatic populations of red and grey squirrels, as deduced by interviewing forest rangers across Northern Ireland, appears similar to the distributions outlined in the Irish Squirrel Survey [17]. Red squirrels, whilst widespread, are typically restricted to forests where only they occur and are isolated or surrounded by forests with grey squirrels or sympatric populations (Figure 2A). It is likely that the distribution of the grey squirrel has been underestimated, as common species are typically reported less often than notably rare species.

Notwithstanding variation in the prevalence of the virus between sampled tissues, the number of grey squirrels tested that were infected with SQPV was relatively low at 8% at the time of sampling. The majority of grey squirrels infected were found in forests with sympatric populations (13 of 18 virus positive grey squirrels). A much larger proportion of squirrels sampled (22%) tested positive for SQPV antibodies. Most of these came from areas of grey squirrel allopatry (34 of 54 antibody positive grey squirrels). The distribution of seropositive squirrels was widespread; most forests with grey squirrels were positive for SQPV antibodies. Indeed, only five of the 37 forests tested had no seropositive samples, suggesting no history of SQPV infection. Of those five seronegative forests, three tested positive for SQPV DNA at the time of sampling, suggesting the virus may have recently spread to these areas.

The presence of grey squirrels with a high prevalence of SQPV poised to invade red squirrel-only habitats poses a substantial risk to remaining red squirrel populations (e.g. Mount Stewart; Figure 2A & C). However, as this study had low sample sizes, we recommend that these effects are reanalysed using a larger sample.

Most squirrels with SQPV DNA were negative for SQPV antibodies, suggesting that at the time of testing they were in the earlier stages of infection before any host antibody immune response could develop. Only one red squirrel examined was positive for SQPV DNA or antibodies. This individual presented a very high viral load, and also had thick scabs on the lower lip and severe dermatitis. No other red squirrels tested positive for SQPV, or SQPV antibodies, which may support the notion that most red squirrels that contract SQPV die from the infection [6]. Equally, it could indicate that prevalence of SQPV in red squirrels is genuinely low. However, it is somewhat disconcerting that some of the red squirrels testing negative were classed as suspected infected, and consequently culled by local authorities prior to being sent to the laboratory for analysis. This suggests that the mechanisms for identifying potential infected live animals is inaccurate (see also [10]) and that culling on this basis alone could, in fact, put extra pressure on already threatened populations. It also highlights the importance of knowing the infection status of sympatric squirrel populations before decisions are taken to cull.

The presence of SQPV antibodies has previously been shown to differ between the sexes and to be most prevalent during autumn/winter [12]. Males typically have larger home ranges than females and travel greater distances, increasing their likelihood of encountering the disease, whilst testosterone may lower their immune response making them more vulnerable to infection [24]–[25]. Our results indicate that the prevalence of the virus was higher in spring/summer and lower in winter. This is complimented by electron microscopy data of SQPV degradation rates, which in part suggests that the virus survived longer in warm, dry conditions than in cool, wet conditions (though there were also more degraded virus particles in warmer, dry conditions). Spring and summer are also likely to favour vector-borne (ectoparasite) transmission. Indeed, several ectoparasitic species examined hosted the virus, including fleas and ticks that occurred on infected squirrels, confirming previous suppositions that they may act as vectors [16], [18], [24].

The presence of viral DNA in the faeces and saliva from the PCR positive red squirrel and its absence from the faeces and saliva of PCR positive grey squirrels suggests that environmental spread of the virus may be a viable route of transmission in red squirrels (due to their high viral load and expression of diarrhoea-like symptoms as observed in the infected red squirrel), but less likely in grey squirrels. The detection of SQPV DNA in the urine of two grey squirrels yet the failure to detect the virus DNA in either the blood or lip tissue is notable, however, rodents are known to pass several viruses in their urine [25]–[26] and SQPV may be passed between squirrels in a similar manner (although SQPV DNA was not observed in SQPV positive red squirrel urine, the sample size was very low, so transmission via this route cannot be discounted). Thus, whilst the most likely vectors for the disease appear to be ectoparasites, we cannot rule out a faecal or saliva route in red squirrels or urine in either species. The potential impact of co-infection with other pathogens (for example, with adenovirus, [27]) on the susceptibility of squirrels to SQPV has not been investigated but should be a key area for future research.

SQPV is indigenous to grey squirrels and was present in most populations throughout the study region. Grey squirrels typically had low viral loads and recovered from the disease. Forests with no history of SQPV infection (i.e. sampled squirrels lacked antibodies) but where the virus was detected, occurred in interface areas between grey and red squirrel populations and pose the greatest risk to nearby red squirrel populations (e.g. in this specific case study; Mount Stewart, Newtownards, Co. Down). Limiting the contact between the two species in these areas may provide one means by which to slow or prevent the spread of the disease to remaining red squirrels. In areas where the ranges of the two species overlap, measures to reduce encounter rates are considered essential [16].

## References

[1] Gurnell J (1996) The grey squirrel in Britain: problems for management and lessons for Europe. In: Mathias ML, Santos-Reis M, Amori G, Libois R, Mitchell-Jones A, et al.. editors. European Mammals: Proceedings of the I European Congress of Mammalogy. Museu Bocage. 67–81.

[2] Wauters LA, Gurnell J (1999) The mechanism of replacement of red squirrels by grey squirrels: A test of the interference competition hypothesis. Ethology 105: 1053–1071.

[3] Gurnell J, Wauters LA, Lurz PW, Tosi G (2004) Alien species and interspecific competition: effects of introduced eastern grey squirrels on red squirrel population dynamics. J Anim Ecol 73: 26–35.

[4] Rushton SP, Lurz PW, Gurnell J, Nettleton P, Bruemmer C, et al (2006) Disease threats posed by alien species: the role of a poxvirus in the decline of the native red squirrel in Britain. Epidemiol Infect 134: 521–533. 16238822 10.1017/S0950268805005303 PMC2870420

[5] McInnes CJ, Wood AR, Thomas K, Sainsbury AW, Gurnell J, et al (2006) Genomic characterization of a novel poxvirus contributing to the decline of the red squirrel (*Sciurus vulgaris*) in the UK. J Gen Virol 87: 2115–2125. 16847106 10.1099/vir.0.81966-0

[6] Sainsbury AW, Nettleton P, Gilray J, Gurnell J (2000) Grey squirrels have high seroprevalence to a parapoxvirus associated with deaths in red squirrels. Anim Conserv 3: 229–233.

[7] Tompkins DM, Sainsbury AW, Nettleton P, Buxton D, Gurnell J (2002) Parapoxvirus causes a deleterious disease in red squirrels associated with UK population declines. Proc R Soc Lond B Biol Sci 269: 529–533. 10.1098/rspb.2001.1897 PMC1690913 11886647

[8] Simpson VR, Hargreaves J, Everest DJ, Baker AS, Booth PA, et al (2010) Mortality in red squirrels (*Sciurus vulgaris*) associated with exudative dermatitis. Vet Rec 167: 59–62. 20622205 10.1136/vr.b4887

[9] Duff JP, Scott A, Keymer IF (1996) Parapox virus infection of the grey squirrel. Vet Rec 138: 527. 8761982

[10] Carroll B, Russell P, Gurnell J, Nettleton P, Sainsbury AW (2009) Epidemics of squirrel pox virus disease in red squirrels (*Sciurus vulgaris*): temporal and serological findings. Epidemiol Infect 137: 257–265. 18606024 10.1017/S0950268808000836

[11] Sainsbury AW, Deaville R, Lawson B, Cooley WA, Farrelly SSJ, et al (2008) Poxviral disease in red squirrels *Sciurus vulgaris* in the UK: spatial and temporal trends of an emerging threat. Ecohealth 5 305–316. 18923872 10.1007/s10393-008-0191-z

[12] Bruemmer CM, Rushton SP, Gurnell J, Lurz PW, Nettleton P, et al (2010) Epidemiology of squirrel pox virus in grey squirrels in the UK. Epidemiol Infect 138: 941–950. 20412610 10.1017/S0950268810000816

[13] University of Liverpool website. Available: https://news.liv.ac.uk/2013/11/15/formbys-red-squirrel-population-recovering/. Accessed 2013 Nov 15.

[14] Middleton AD (1930) The ecology of the American grey squirrel (*Sciurus carolinensis*) in the British Isles. Proc Zool Soc Lond 2: 809–843.

[15] Scott AC, Keymer IF, Labram J (1981) Parapoxvirus infection of the red squirrel (*Sciurus vulgaris*). Vet Rec 109: 202. 6275598 10.1136/vr.109.10.202

[16] McInnes CJ, Coulter L, Dagleish MP, Deane D, Gilray J, et al (2012) The emergence of squirrel pox in Ireland. Anim Conserv 16: 51–59.

[17] Carey M, Hamilton G, Poole A, Lawton C (2007) The Irish Squirrel Survey. COFORD, Dublin.

[18] Atkin JW, Radford AD, Coyne KP, Stavisky J, Chantrey J (2010) Detection of squirrel poxvirus by nested and real-time PCR from red (*Sciurus vulgaris*) and grey (*Sciurus carolinensis*) squirrels. BMC Vet Res 6: 33. 20529323 10.1186/1746-6148-6-33 PMC2887832

[19] Gurnell J, Lurz PWW, McDonald RA, Pepper H (2009) Practical techniques for surveying and monitoring squirrels. Forestry Commission Practice Note No.11.

[20] Lees J (2011) Control of grey squirrels for red squirrel conservation - a code of good practice. Northern Ireland Squirrel Forum Standard Operating Procedure.

[21] Sweet D, Lorente M, Lorente JA, Valenzuela A, Villanueva E (1997) An improved method to recover saliva from human skin: the double swab technique. J Forensic Sci 42: 320–322. 9068193

[22] Livingston CW, Hardy WT (1960) Longevity of contagious ecthyma virus. J Am Vet Med Assoc 137: 651.

[23] Everest DJ, Stidworthy MF, Milne EM, Meredith AL, Chantrey J, et al (2010) Retrospective detection by negative contrast electron microscopy of faecal viral particles in wild red squirrels (*Sciurus vulgaris*) with suspected enteropathy in Great Britain. Vet Rec 167: 1007–1010. 21262731 10.1136/vr.c4111

[24] LaRose JP, Meredith AL, Everest DJ, Fiegna C, McInnes CJ, et al (2010) Epidemiological and postmortem findings in 262 red squirrels (*Sciurus vulgaris*) in Scotland, 2005 to 2009. Vet Rec 167: 297–302. 20729517 10.1136/vr.c4196

[25] Bharti AR, Nally JE, Ricaldi JN, Matthias MA, Diaz MM, et al (2003) Leptospirosis: a zoonotic disease of global importance. Lancet Infect Dis 3: 757–771. 14652202 10.1016/s1473-3099(03)00830-2

[26] Kallio ER, Klingstrom J, Gustafsson E, Manni T, Vaheri A, et al (2006) Prolonged survival of *Puumala hantavirus* outside the host: evidence for indirect transmission via the environment. J Gen Virol 87: 2127–2134. 16847107 10.1099/vir.0.81643-0

[27] Everest DJ, Griffin J, Warnock ND, Collins LM, Dick J, et al (2012) Adenovirus particles from a wild red squirrel (*Sciurus vulgaris*) from Northern Ireland. Vet Rec 170: 188. 22354657 10.1136/vr.e1128

